# High-frequency ultrasound combined with superb microvascular imaging for differential diagnosis of basal cell carcinoma and seborrheic keratosis: a retrospective analysis with nomogram implementation

**DOI:** 10.3389/fmed.2026.1815897

**Published:** 2026-05-12

**Authors:** Guiwu Chen, Xiaoling Leng, Wenqin Liu, Xiaomin Liao, Yuting Li, Jiaxin Meng, Yongjie Cao

**Affiliations:** 1 Department of Ultrasound, The Tenth Affiliated Hospital, Southern Medical University (Dongguan People’s Hospital), Dongguan, China; 2 Department of Pathology, The Tenth Affiliated Hospital, Southern Medical University (Dongguan People’s Hospital), Dongguan, China; 3 Department of Radiology, The Tenth Affiliated Hospital, Southern Medical University (Dongguan People’s Hospital), Dongguan, China

**Keywords:** basal cell carcinoma, differential diagnosis, high-frequency ultrasound, nomogram, seborrheic keratosis, superb microvascular imaging

## Abstract

**Objective:**

To evaluate the diagnostic performance of high-frequency ultrasound (HFUS) combined with superb microvascular imaging (SMI) in differentiating basal cell carcinoma (BCC) from seborrheic keratosis (SK), and to develop a nomogram for individualized probability estimation.

**Methods:**

This retrospective analysis included 208 patients (49 BCC, 159 SK) with histopathologically confirmed lesions and complete preoperative HFUS and SMI records. Grayscale ultrasound assessed morphological features (size, depth, shape, margins, echogenicity, etc.), while SMI evaluated microvascular flow patterns and grading. Univariate and multivariate logistic regression analyses identified independent predictors, and a nomogram was constructed to estimate the probability of BCC.

**Results:**

Univariate analysis revealed significant differences between BCC and SK in terms of age, lesion location, size, depth, morphology, internal echotexture, posterior acoustic features, and microvascular flow patterns (all *p* < 0.001). Multivariate logistic regression analysis identified larger tumor size, head location, nodular ultrasound pattern, and random vascular flow as independent predictors of BCC. The resulting multivariate logistic regression model demonstrated good discriminative performance, with an area under the receiver operating characteristic curve (AUC) of 0.861. At the optimal cutoff, the model yielded a sensitivity of 77.55% and a specificity of 82.38%. A nomogram was subsequently constructed based on these independent predictors to facilitate individualized probability estimation of BCC in clinical practice.

**Conclusion:**

HFUS combined with SMI provides a valuable non-invasive approach for differentiating BCC from SK. The developed nomogram, based on key ultrasound and clinical predictors, demonstrates good discriminative performance and offers a practical tool to support clinical decision-making, potentially reducing unnecessary biopsies and facilitating earlier detection of malignant lesions.

## 1 Introduction

Basal cell carcinoma (BCC) and seborrheic keratosis (SK) are two commonly encountered skin lesions in dermatological practice, each with distinct biological behaviors and clinical implications (1). BCC, the most prevalent form of skin cancer, originates from the basal layer of the epidermis and is known for its locally invasive growth pattern, albeit with a low metastatic potential (2). In contrast, SK is a benign epidermal tumor, often referred to as “senile wart” or “basal cell papilloma,” characterized by its benign nature and typical appearance in elderly individuals (3).

The clinical differentiation between BCC and SK can be challenging due to their overlapping morphological features, particularly in their early stages or when located in sun-exposed areas such as the head and face (4, 5). Misdiagnosis can lead to inappropriate treatment strategies, potentially compromising patient outcomes and increasing healthcare costs. Therefore, accurate and timely diagnosis is crucial for effective management and optimal patient care.

Traditionally, skin biopsy followed by histopathological examination has been the gold standard for diagnosing skin lesions. However, this invasive procedure is associated with potential complications, including scarring and infection, and may not be suitable for all patients, especially those with multiple lesions or those who prefer non-invasive diagnostic methods (6–8). In current practice, dermoscopy is widely used as a non-invasive initial assessment tool, offering detailed visualization of surface structures and pigmentation patterns (4, 9, 10). However, dermoscopy remains problematic for distinguishing BCC from SK due to overlapping pigmented features and altered vascular visibility, especially in skin of color (11). It is also limited in evaluating deep morphological features such as tumor thickness, infiltration depth, and internal vascular architecture.

High-frequency ultrasound (HFUS) has emerged as a promising non-invasive tool for the evaluation of skin lesions. With its ability to provide high-resolution images of skin structures, HFUS can clearly delineate the anatomical details of skin tumors, including their size, depth, margins, and internal vascularity (12, 13). This information is invaluable for differentiating benign from malignant lesions and for guiding treatment decisions. Superb microvascular imaging (SMI) is a novel ultrasound technique that enhances the visualization of microvascular blood flow within tissues (14). By providing detailed maps of tumor vascularity, SMI can further improve the diagnostic accuracy of HFUS, particularly in cases where the distinction between benign and malignant lesions is subtle (15, 16). Therefore, integrating HFUS and SMI into the diagnostic workflow may be particularly valuable in cases where dermoscopic findings are equivocal or when deeper tissue characterization is required.

In this context, we conducted a 18-year retrospective analysis to evaluate the efficacy of HFUS combined with SMI in the differential diagnosis of BCC and SK. Our study aims to provide comprehensive insights into the diagnostic performance of this combined imaging approach, with the ultimate goal of enhancing clinical decision-making and improving patient outcomes. Through this research, we hope to contribute to the growing body of evidence supporting the use of non-invasive imaging techniques in dermatology and to pave the way for more widespread adoption of HFUS and SMI in the clinical setting, particularly in cases where dermoscopy is inconclusive.

## 2 Materials and methods

### 2.1 Study design and patient selection

**Figure 1:**
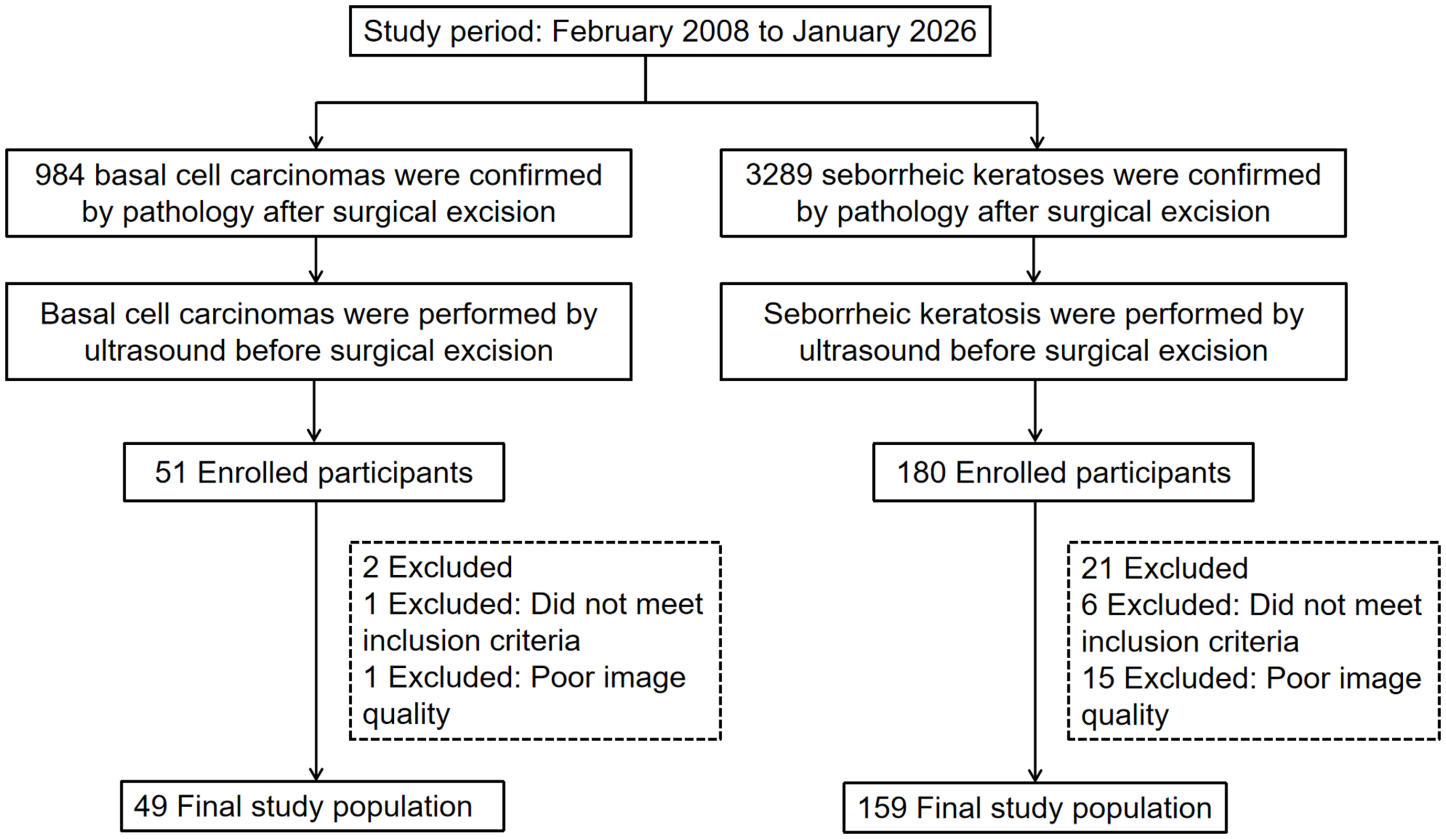
Patient flowchart of basal cell carcinoma and seborrheic keratosis.

**Figure 2:**
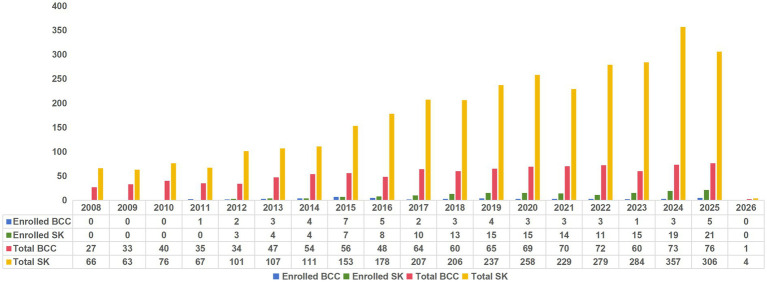
Bar chart of included cases versus total cases.

This retrospective analysis utilized data extracted from the Pathology Report System of Dongguan People’s Hospital, covering the period from February 2008 to January 2026. A total of 4,273 patients with surgical resection and pathological confirmation were initially identified, comprising 984 BCC and 3,289 SK cases. After applying inclusion and exclusion criteria, 208 patients (49 BCC, 159 SK) with complete preoperative HFUS and SMI records in the Picture Archiving and Communication System (PACS) constituted the final study cohort (Figures 1, 2).

Inclusion required: (1) histopathologically verified BCC or SK; (2) availability of complete preoperative ultrasound; (3) no prior treatment or intervention at the lesion site before ultrasound scanning. Exclusion criteria: (1) incomplete clinical, pathological, or imaging documentation; (2) any history of therapeutic intervention (e.g., surgery, cryotherapy) preceding ultrasound; (3) lesions with extensive surface ulceration or crusting that compromised adequate acoustic coupling.

### 2.2 Grayscale ultrasound and morphological analysis

Grayscale ultrasound examinations were performed using a Canon Aplio i900 ultrasound system (Canon Medical Systems Corporation) equipped with an i18LX5 linear transducer (frequency range: 5 to 18 MHz). Patients were appropriately positioned to fully expose the target lesion, and adequate ultrasound gel was applied to ensure optimal acoustic coupling. Scanning parameters—including depth, gain, focal zone, and frequency—were optimized to clearly delineate the lesion’s borders, depth of invasion, and its relationship to adjacent anatomical structures. Representative images in both longitudinal and transverse planes were archived in PACS.

**Figure 3:**
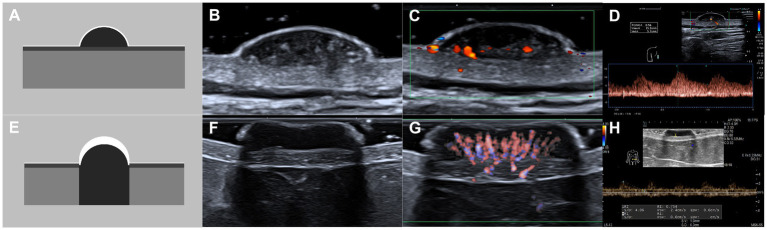
Schematic illustrations and ultrasound patterns of seborrheic keratosis. **(A–D)** Superficial pattern: regular shape, well-defined margin, homogeneous echo, and typically presenting with a flat basal surface that does not invade the dermis. **(A)** Schematic illustration, **(B)** grayscale ultrasound, **(C)** color Doppler flow imaging, **(D)** pulsed wave Doppler. **(E–H)** Superficial pattern with posterior acoustic shadowing: regular shape, well-defined margin, homogeneous echo, posterior acoustic shadowing and often presenting with pronounced hyperkeratosis on the surface and a flat basal surface that does not invade the dermis. **(E)** Schematic illustration, **(F)** grayscale ultrasound, **(G)** superb microvascular imaging, **(H)** pulsed wave Doppler.

**Figure 4:**
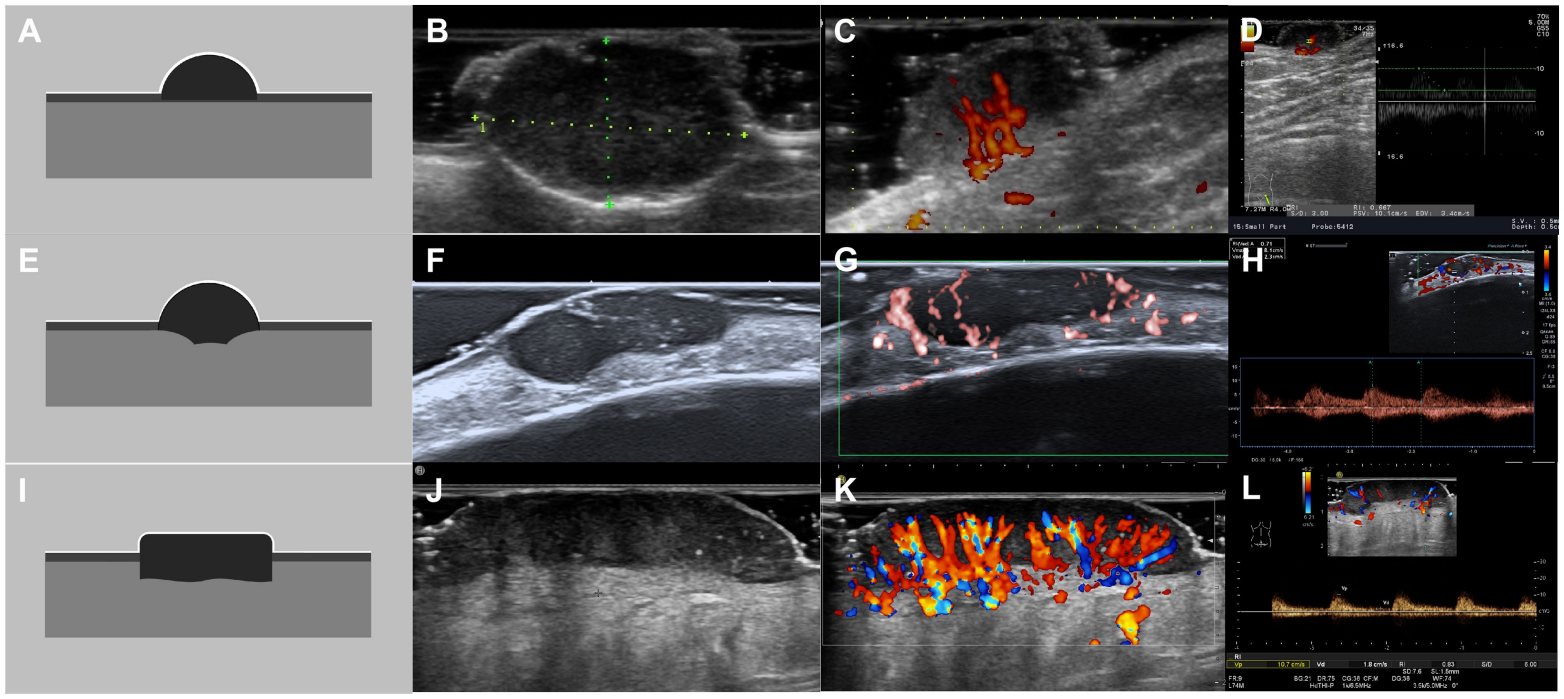
Schematic illustrations and ultrasound patterns of basal cell carcinoma. **(A–D)** Superficial pattern: regular shape, well-defined margin, homogeneous echo, and typically presenting with a flat basal surface with minimal or no dermal involvement. **(A)** Schematic illustration, **(B)** grayscale ultrasound, **(C)** superb microvascular imaging, **(D)** pulsed wave Doppler. **(E–H)** Nodular pattern: irregular shape, well-defined margin, homogeneous or heterogeneous echo, and often presenting with an uneven basal surface that partially invades the dermis. **(E)** Schematic illustration, **(F)** grayscale ultrasound, **(G)** superb microvascular imaging, **(H)** pulsed wave Doppler. **(I–L)** Infiltrative pattern: irregular shape, well-defined or ill-defined margin, homogeneous or heterogeneous echo, and often presenting with an uneven basal surface that mostly invades the dermis, involving the subcutaneous tissue and even muscle. **(I)** schematic illustration, **(J)** grayscale ultrasound, **(K)** color Doppler flow imaging, **(L)** pulsed wave Doppler.

Two experienced sonologists, blinded to pathological results, independently evaluated the grayscale images. The following morphological features were assessed: size (maximum diameter in mm); depth (mm from the skin surface); shape (regular or irregular); margins (well-defined or ill-defined); internal echogenicity (hypoechoic or hyperechoic relative to adjacent dermis); homogeneity (homogeneous or heterogeneous); hyperechoic foci (yes or no; suggestive of keratin or microcalcifications); anechoic area (yes or no); posterior acoustic features (no changed, attenuated, or enhanced); based on these features, the lesions were further classified into one of the following ultrasound patterns: superficial, nodular, or infiltrative (Figures 3, 4) (17).

### 2.3 Superb microvascular imaging and vascular analysis

Following grayscale imaging, microvascular flow was assessed using the SMI mode on the same ultrasound system. SMI employs advanced clutter suppression and motion artifact reduction algorithms to visualize low-velocity microvascular flow with high spatial resolution. Imaging settings—including velocity scale, gain, and wall filter—were optimized with specific settings: velocity scale (pulse repetition frequency) was set at −5.0 to 5.0 cm/s, gain was adjusted to 60 to 80% (just below the noise threshold), and the wall filter was kept at the lowest available setting to maximize sensitivity while minimizing background noise. Light transducer pressure was maintained to avoid vascular compression. Longitudinal and transverse SMI images were stored in the PACS for blinded evaluation.

The same two reviewers independently analyzed SMI images with a focus on the following vascular characteristics. Flow abundance was graded according to a modified Adler scale: Grade 0 indicates no detectable flow; Grade 1 indicates minimal flow (1 to 2 dot-like or short linear signals); Grade 2 indicates moderate flow (3 to 4 signals or one main vessel with branches); and Grade 3 indicates marked flow (5 or more than 5 signals or multiple branching vessels). Based on the flow abundance, vascular distribution pattern was categorized as follows: absent, no flow signal; peripheral, where flow signals are predominantly around the lesion periphery; arborizing, characterized by linear vessels extending into the lesion center with branching; or random, featuring irregularly distributed dot-like or short vascular signals (18).

### 2.4 Statistical analysis

Data analysis was performed using SPSS 26.0 (IBM Corp). Continuous variables were expressed as mean ± standard deviation and compared between groups with independent *t*-tests. Categorical variables were presented as frequencies and percentages, and group comparisons were conducted using chi-square tests. Key independent variables were selected based on clinical relevance and entered into a multivariate logistic regression model (forward stepwise method) to identify independent predictors, after assessing multicollinearity through variance inflation factors (VIF > 5 indicated significant collinearity). Odds ratios with 95% confidence intervals were reported. A two-tailed *p*-value < 0.05 was considered statistically significant. The discriminative performance of the final multivariate logistic regression model was assessed using the area under the receiver operating characteristic curve (AUC-ROC). A predictive nomogram was developed based on the final multivariate logistic regression model.

## 3 Results

### 3.1 Univariate analysis of patient demographics, clinical characteristics, and ultrasound features

A total of 4,273 patients with surgical resection and pathological confirmation were initially identified, comprising 984 BCC and 3,289 SK cases during February 2008 to January 2026. Comparison between included and total cases revealed no significant differences in age, gender, or tumor location distribution (all *p* > 0.05) except for SK tumor location distribution was observed (*p* = 0.017) (Table 1). Two hundred eight patients were included in the final analysis, comprising 49 cases of BCC and 159 cases of SK. Univariate analysis revealed significant differences between the two groups across several demographic, clinical, and ultrasound parameters. Patients with BCC were significantly older than those with SK (65.10 ± 12.66 years *vs*. 56.70 ± 15.26 years, *p* < 0.001). Gender distribution was comparable between groups. Tumor location differed markedly: BCC lesions predominantly occurred on the head (77.55%), whereas SK lesions were more widely distributed across the head (35.84%), trunk (30.18%), limbs (20.75%), neck (8.81%), and girdle region (4.40%) (Table 2).

**Table 1:** Included cases versus total cases of basal cell carcinoma and seborrheic keratosis.

Parameters	Basal cell carcinoma		*t/χ^2^*	*p*	Seborrheic keratosis		*t/χ^2^*	*p*
	Included	Total			Included	Total		
Age (year)	65.10 ± 12.66	64.27 ± 13.91	0.395	0.693	56.70 ± 15.26	54.03 ± 16.05	1.945	0.052
Gender			0.655	0.418			0.689	0.406
Male	25	444			81	1786		
Female	24	540			78	1,503		
Tumor location			3.650	0.455			12.098	0.017
Head	38	829			57	1,565		
Neck	0	13			14	196		
Girdle	1	15			7	201		
Trunk	7	75			48	843		
Limb	3	52			33	484		

**Table 2:** Univariate analysis of patient demographics, clinical characteristics, and ultrasound features.

Parameters	Total	Basal cell carcinoma	Seborrheic keratosis	*t/χ^2^*	*p*
Age (year)	58.68 ± 15.09	65.10 ± 12.66	56.70 ± 15.26	3.858	<0.001
Gender				<0.001	0.992
Male	106	25	81		
Female	102	24	78		
Tumor location				27.336	<0.001
Head	95	38	57		
Neck	14	0	14		
Girdle	8	1	7		
Trunk	55	7	48		
Limb	36	3	33		
Size (mm)	12.76 ± 7.19	16.39 ± 8.41	11.64 ± 6.41	3.634	0.001
Depth (mm)	4.47 ± 2.76	6.37 ± 3.37	3.88 ± 2.26	4.839	<0.001
Shape				28.743	<0.001
Regular	148	20	128		
Irregular	60	29	31		
Margin				21.717	<0.001
Well-defined	161	26	135		
Ill-defined	47	23	24		
Internal echogenicity					
Hypoechoic	202	49	153	1.904	0.168
Hyperechoic	6	0	6		
Homogeneity				33.191	<0.001
Homogeneous	128	13	115		
Heterogeneous	80	36	44		
Hyperechoic foci				24.806	<0.001
Yes	28	17	11		
No	180	32	148		
Anechoic area				13.142	<0.001
Yes	12	8	4		
No	196	41	155		
Posterior acoustic features				35.102	<0.001
No change	107	31	76		
Attenuation	72	2	70		
Enhancement	29	16	13		
Ultrasound patterns				21.602	<0.001
Superficial	115	13	102		
Nodular	79	30	49		
Infiltrative	14	6	8		
Blood flow patterns				31.264	<0.001
Absent	130	18	112		
Arborizing	42	10	32		
Peripheral	6	4	2		
Random	30	17	13		
Blood flow grading				18.504	<0.001
Grade 0	130	18	112		
Grade 1	20	7	13		
Grade 2	27	11	16		
Grade 3	31	13	18		

Significant ultrasound distinctions were also observed. BCC lesions exhibited a larger maximum diameter (16.39 ± 8.41 mm *vs*. 11.64 ± 6.41 mm, *p* = 0.001) and greater depth of invasion (6.37 ± 3.37 mm *vs*. 3.88 ± 2.26 mm, *p* < 0.001). Morphologically, BCC lesions were more frequently irregular in shape (59.18% vs. 19.49%, *p* < 0.001) and had ill-defined margins (46.94% vs. 15.09%, *p* < 0.001). Internal echo texture was more often heterogeneous in BCC (73.47% vs. 27.67%, *p* < 0.001), and the presence of internal punctate hyperechoic foci (34.69% vs. 6.92%, *p* < 0.001) and anechoic areas (16.33% vs. 2.52%, *p* < 0.001) was more common. Posterior acoustic features also differed: enhancement was more frequent in BCC (32.65%), while attenuation predominated in SK (44.03%). The nodular ultrasound pattern was most common in BCC (61.22%), whereas the superficial pattern predominated in SK (64.15%).

Microvascular flow assessment demonstrated further differentiation. Absent or minimal flow (Adler Grade 0–1) was more common in SK (78.62%), while BCC lesions more frequently exhibited moderate to marked flow (Grade 2–3) (48.97%). Arborizing and random vascular patterns were more frequently associated with BCC, whereas absent flow was more typical of SK. All these ultrasound and vascular characteristics showed statistically significant differences between BCC and SK (all *p* < 0.001).

### 3.2 Multivariate analysis of patient demographics, clinical characteristics, and ultrasound features

Based on the multivariate logistic regression analysis, several ultrasound and clinical features were identified as independent predictors for distinguishing BCC from SK. After adjusting for potential confounders, lesion size remained a significant predictor, with each 1 mm increase in maximum diameter associated with a 9% higher likelihood of BCC (*OR* = 1.09, 95% *CI*: 1.01–1.176, *p* = 0.027). Tumor location also demonstrated strong predictive value: compared to lesions located on the head, those on the trunk (*OR* = 0.201, 95% *CI*: 0.067–0.603, *p* = 0.004) and limbs (*OR* = 0.094, 95% *CI*: 0.023–0.396, *p* = 0.001) were significantly less likely to be BCC (Table 3).

**Table 3:** Multivariate analysis of patient demographics, clinical characteristics, and ultrasound features.

Parameters	*B*	SE	*Wals*	*df*	*p*	OR	95% CI	
							Lower limit	Upper limit
Age (year)	0.004	0.017	0.049	1	0.825	1.004	0.971	1.037
Size (mm)	0.086	0.039	4.912	1	0.027	1.090	1.010	1.176
Location			16.305	4	0.003			
Neck	−21.336	N/E	N/E	1	0.998	N/E	N/E	N/E
Trunk	−1.603	0.560	8.201	1	0.004	0.201	0.067	0.603
Girdle	−2.810	1.548	3.295	1	0.069	0.060	0.003	1.251
Limb	−2.359	0.731	10.409	1	0.001	0.094	0.023	0.396
Ultrasound patterns			8.004	2	0.018			
Nodular	1.247	0.443	7.932	1	0.005	3.478	1.461	8.281
Infiltrative	0.594	0.888	0.449	1	0.503	1.812	0.318	10.320
Blood flow patterns			7.969	3	0.047			
Random	1.229	0.575	4.566	1	0.033	3.419	1.107	10.559
Arborizing	−0.085	0.557	0.023	1	0.878	0.918	0.308	2.734
Peripheral	2.372	1.314	3.260	1	0.071	10.721	0.816	140.811
Constant	−2.629	0.961	7.480	1	0.006	0.072		

N/E, not estimable.

Among the ultrasound morphological patterns, the nodular pattern emerged as an independent predictor for BCC compared to the superficial pattern (*OR* = 3.478, 95% *CI*: 1.461–8.281, *p* = 0.005). In terms of microvascular flow characteristics, the random pattern was significantly associated with BCC (*OR* = 3.419, 95% *CI*: 1.107–10.559, *p* = 0.033). Although the peripheral flow pattern showed a strong positive association with BCC (*OR* = 10.721), it did not reach conventional statistical significance (*p* = 0.071). Other patterns, including arborizing flow pattern, did not exhibit significant independent predictive value.

Notably, patient age—which was significantly different between groups in univariate analysis—was not retained as an independent predictor in the multivariate model (*p* = 0.825). Similarly, the infiltrative ultrasound pattern and several other ultrasound features did not demonstrate significant independent associations after adjustment. These results highlight that a combination of size, location, nodular ultrasound pattern, and random vascularity provides a more robust multivariate discriminative profile for BCC versus SK than age or other morphological features alone.

**Figure 5:**
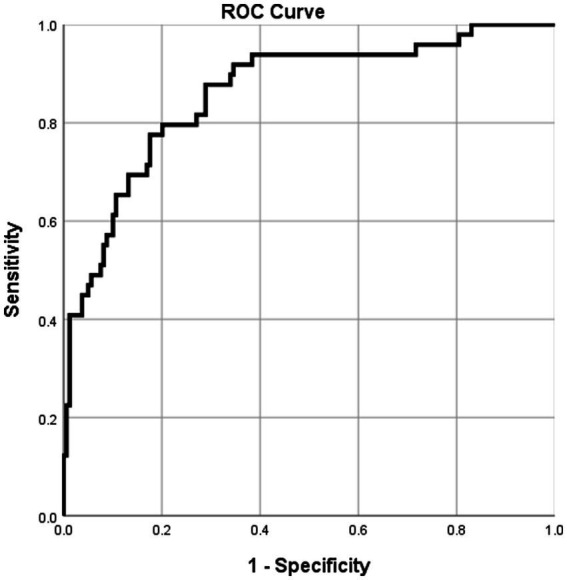
ROC curve of the multivariate model.

The discriminative performance was evaluated using ROC curve analysis. The AUC was 0.861, indicating good diagnostic accuracy in differentiating BCC from SK. The optimal cutoff value was determined using the Youden index, yielding a sensitivity of 77.55% and a specificity of 82.38% (Table 4 and Figure 5).

**Table 4:** Diagnostic performance of the multivariate model.

Parameter	Result
Sensitivity	77.55%
Specificity	82.38%
False positive rate	17.62%
False negative rate	22.45%
Accuracy	81.25%
Prevalence	23.56%
Positive predictive value	57.58%
Negative predictive value	92.25%
Positive likelihood ratio	4.40
Negative likelihood ratio	0.27
Diagnostic odds ratio	16.16
Kappa	0.54

### 3.3 Predictive model development and application

**Figure 6:**
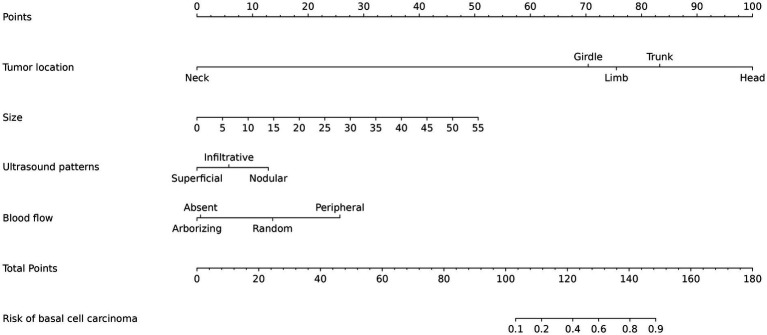
Nomogram of basal cell carcinoma and seborrheic keratosis.

Based on the independent predictors identified in the multivariate logistic regression analysis, a nomogram was constructed to estimate the individualized probability of BCC versus SK. The nomogram incorporated the following significant variables: tumor size, location, ultrasound pattern, and blood flow pattern. Each variable was assigned a points scale proportional to its regression coefficient, allowing for the summation of total points corresponding to the predicted probability of BCC (Figure 6). To illustrate the application of the nomogram, consider the following cases:

Case 1: 65-year-old female, head lesion, 11 mm, nodular ultrasound pattern, random blood flow pattern, nomogram score 139 = 100 + 10 + 14 + 15, malignant probability approximately 0.78, immediate biopsy recommended.

Case 2: 48-year-old female, trunk lesion, 17 mm, superficial ultrasound pattern, no obvious blood flow, nomogram score 98 = 83 + 15 + 0 + 0, malignant probability approximately 0.05, benign management recommended.

Case 3: 77-year-old male, trunk lesion, 11 mm, superficial ultrasound pattern, peripheral blood flow pattern, nomogram score 118 = 83 + 10 + 0 + 25, malignant probability 0.30, close monitoring recommended.

## 4 Discussion

### 4.1 Associated factors identified in univariate analysis of basal cell carcinoma and seborrheic keratosis

In the univariate analysis comparing BCC and SK, several factors demonstrated meaningful associations with these dermatological conditions, encompassing patient demographics, clinical presentation, and ultrasound attributes.

Patients diagnosed with BCC were notably older than those with SK, consistent with the understanding that BCC often emerges in later decades of life, likely due to cumulative sun exposure over time. In contrast, SK, although frequent in older adults, can appear at a relatively younger age, possibly reflecting different pathways in skin aging or genetic predisposition (19). No significant difference was observed between genders for either condition, supporting the view that both lesions develop irrespective of sex, an observation that aligns with broader epidemiological patterns in dermatology.

Regarding clinical characteristics, tumor location differed markedly between the two entities. BCC arose most often on the head, underscoring its strong link to chronic ultraviolet radiation exposure on sun-exposed skin (20). On the other hand, SK demonstrated a wider anatomical distribution, involving not only the head but also the trunk and limbs in considerable proportions, suggesting that its development may be tied to more systemic or multifactorial processes of skin maturation rather than sun exposure alone.

Ultrasound evaluation further highlighted distinct features between the two lesions. BCC generally presented with larger dimensions and greater invasion depth compared to SK, reflecting its locally aggressive and infiltrative biological behavior (21). HFUS clearly delineates skin layers, revealing that SK is typically confined to the epidermis, often with a flat base resembling a “flat bottom” sign, whereas BCC frequently involves the dermis or subcutaneous tissue (22). Morphologically, BCC more frequently exhibited irregular contours and poorly defined margins, characteristics that correspond to its invasive growth pattern, whereas SK typically displayed regular shapes and clearer boundaries (23). Internally, BCC lesions often showed heterogeneous echogenicity along with punctate echogenic foci and anechoic areas, which may correlate with microcalcifications, keratin deposits, or small necrotic zones within the tumor (24). Notably, similar punctate hyperechoic foci can also be observed in irritated SK, necessitating careful differentiation. In contrast, SK was predominantly homogeneous in echo texture with fewer internal echogenic or anechoic findings. It is important to note that marked surface hyperkeratosis in some SK lesions can cause significant posterior acoustic attenuation, potentially obscuring visualization of the lesion base (25). Additionally, during examination, gentle transducer pressure should be maintained to avoid compression induced deformation of the typically flat-based SK. Posterior acoustic features also varied, with enhanced through transmission being more common in BCC, possibly due to structural changes in tissue density and interface properties (26). Furthermore, some SK lesions may appear quite thick on ultrasound but remain strictly epidermal without dermal or subcutaneous involvement, a feature helpful in distinguishing them from invasive BCC.

Microvascular assessment revealed that BCC was associated with more pronounced and complex vascular patterns, indicating higher angiogenic activity, while SK mostly presented with absent or minimal blood flow. However, some clinical subtypes of SK, such as pigmented, irritated, and clonal variants, may exhibit rich vascularity on blood flow imaging, which may be associated with inflammation, irritation, or rapid growth, and may be misinterpreted as BCC. These differences in vascularity further underscore the fundamental metabolic and proliferative distinctions between malignant and benign keratinocytic lesions.

In summary, this univariate analysis delineates several differentiating factors between BCC and SK across demographic, clinical, and ultrasound domains. These observations, complemented by practical ultrasound experience, deepen the pathological and clinical understanding of both conditions. Importantly, the ultrasound characteristics demonstrated considerable utility in distinguishing between the two entities, supporting the potential role of ultrasound as a non invasive diagnostic tool in dermatology. Such findings not only aid in clinical decision making but also pave the way for further research into imaging based diagnostic approaches.

### 4.2 Independent predictors from multivariate analysis of basal cell carcinoma and seborrheic keratosis

In the multivariate logistic regression analysis, several key predictors were identified as independently distinguishing BCC from SK. These findings hold significant implications for accurate diagnosis and differentiation of these two common skin lesions in clinical practice.

Firstly, tumor size emerged as a significant independent predictor. Larger tumors were more likely to be BCC rather than SK, aligning with the biological characteristics of BCC as a malignant skin tumor with a generally faster growth rate and higher invasiveness compared to SK, a benign epidermal lesion. This observation suggests that clinicians should be more vigilant about the possibility of BCC when encountering larger skin lesions and consider further diagnostic evaluations (27).

Secondly, lesion location was also identified as an important independent predictor. BCC demonstrated a higher propensity for occurring on the head, whereas SK was more widely distributed across the head, trunk, and limbs (28). This discrepancy in lesion location reflects the distinct etiological and epidemiological patterns of BCC and SK, potentially influenced by factors such as ultraviolet exposure, skin type, and genetic predisposition. Therefore, information regarding lesion location can provide valuable clues for differential diagnosis in clinical settings.

Among the ultrasonic morphological features, the nodular ultrasound pattern was recognized as an independent predictor for BCC. Compared to the superficial pattern, the nodular pattern was more frequently observed in BCC cases. This ultrasonic characteristic likely correlates with the growth pattern and histological features of BCC, which often presents as deeper, well circumscribed nodules. In contrast, SK typically manifests as flatter lesions. Thus, the identification of ultrasonic patterns can aid clinicians in more accurately assessing the nature of skin lesions (29).

Furthermore, blood flow pattern is also an independent predictor for distinguishing between BCC and SK. Specifically, BCC exhibits more abundant vascularity, with blood flow entering from both the base and the periphery of the lesion. This pattern reflects the infiltrative growth of BCC, where tumor cells activate and incorporate adjacent venules, creating a peripheral feeding rim. Consequently, BCC vessels show larger bending angles, greater tortuosity, and marked irregularity. In contrast, SK generally presents with absent or sparse blood flow. When detectable, vessels enter from a flat base and follow a straight course with minimal bending angles. Histologically, this corresponds to blood flow confined within elongated, narrowed dermal papillae, lacking collateral or neovascular networks. Therefore, evaluation of microvascular flow patterns can serve as an adjunctive tool for differentiating BCC from SK (30).

In conclusion, the multivariate analysis revealed that tumor size, lesion location, ultrasonic patterns, and microvascular blood flow patterns are independent predictors for distinguishing BCC from SK. These findings provide clinicians with practical diagnostic tools to enhance the accuracy of differential diagnosis between BCC and SK, thereby guiding more appropriate treatment decisions. Future research could further explore the biological mechanisms underlying these predictors and assess their applicability across diverse populations and clinical settings.

### 4.3 Clinical utility and diagnostic performance of the nomogram for differentiating basal cell carcinoma and seborrheic keratosis

In routine practice, the nomogram provides a structured framework for synthesizing imaging and clinical findings, assisting clinicians in prioritizing lesions for biopsy or planning appropriate management (31, 32). The nomogram developed in this study integrates key ultrasound and clinical predictors—including tumor size, location, ultrasound pattern, and microvascular flow pattern—into a practical, visual scoring tool that estimates the individualized probability of BCC versus SK. With an area under the receiver operating characteristic curve of 0.861, the model demonstrates good discriminative ability, offering a sensitivity of 77.55% and a specificity of 82.38% at the optimal cutoff. These performance metrics suggest that the nomogram can serve as a valuable adjunct in clinical decision-making, particularly when biopsy is not immediately feasible or when non-invasive diagnostic guidance is preferred.

Therefore, integrating high-frequency ultrasound and superb microvascular imaging into the diagnostic workflow is particularly valuable in the following scenarios: (1) when dermoscopic findings are equivocal, for instance in deeply pigmented or inflamed lesions with obscured classic vascular patterns, or in dark-skinned individuals where melanin deposition limits visualization, making differential diagnosis challenging; (2) when precise measurement of tumor thickness or assessment of subcutaneous infiltration depth is required to guide surgical margins or determine suitability for non-surgical treatment; and (3) when a lesion is located in a special anatomical site such as the auricle, nasal ala, or periorbital region, necessitating evaluation of deep structure involvement including muscle, cartilage or bone cortex.

In routine practice, the nomogram provides a structured framework for synthesizing these imaging and clinical findings. Integration of the nomogram into the ultrasound workflow could streamline diagnostic pathways, reduce unnecessary invasive procedures, and support earlier detection of malignant lesions (33, 34). The tool reinforces several clinically relevant patterns: lesions located on the head, especially larger ones, should raise suspicion for BCC, given its strong association with sun-exposed areas and its tendency for greater size and depth. In contrast, smaller lesions situated on the neck, trunk, or limbs are more indicative of SK, which tends to be more broadly and superficially distributed. Importantly, in cases where BCC is small or early-stage—situations in which clinical and dermoscopic differentiation can be challenging—the combination of high-frequency ultrasound and superb microvascular imaging provides enhanced resolution of morphological and vascular details. This multimodal approach improves the detection of subtle features such as irregular margins, heterogeneous echotexture, and random microvascular patterns, thereby aiding in the distinction from benign SK lesions that often exhibit minimal or absent flow.

### 4.4 Limitations and future perspectives

This study has several limitations that should be acknowledged. First, the marked reduction from 4,273 to 208 cases stems from clinical practice patterns at our institution. Dermoscopy remains the first-line non-invasive tool for skin lesions, and HFUS with SMI is not routinely integrated into preoperative assessment. Over the 18-year period, ultrasound was typically performed only when dermoscopic findings were equivocal or when deeper tissue characterization was needed for surgical planning. Consequently, many pathologically confirmed cases lacked preoperative HFUS and SMI. Additionally, in some early cases, some clinicians did not proceed to SMI after observing flow on conventional Doppler. These practice patterns, rather than systematic patient selection, explain the discrepancy between total and included cases. During this study period, advances in ultrasound platforms and SMI software versions over time may have introduced subtle variations in low-velocity flow sensitivity. This potential variability should be considered when interpreting the vascular findings.

Furthermore, although dermoscopy is widely established as the first-line non-invasive diagnostic tool for the evaluation of skin lesions, this study did not include dermoscopic data for direct comparison with HFUS and SMI, as dermoscopic images were not routinely captured or archived in our PACS as part of standard preoperative assessment. Moreover, while dermoscopy serves as the primary diagnostic method, HFUS, particularly when combined with SMI, may play a valuable adjunctive or second-line role in selected cases, offering additional information on tumor depth, vascular architecture, and subsurface morphology. Future prospective studies integrating both dermoscopy and HFUS with SMI are warranted to explore their combined diagnostic performance and to clarify how these modalities may complement each other in a tiered diagnostic approach.

Looking ahead, future studies should focus on prospective validation across diverse clinical settings and populations to confirm the nomogram’s generalizability and refine its diagnostic thresholds. Further research could also explore integrating additional imaging biomarkers or artificial intelligence-assisted features to enhance predictive accuracy (35, 36). In the long term, embedding this tool into point-of-care ultrasound systems may facilitate real-time risk stratification and support standardized reporting (37). By promoting consistent, image-based assessment, the nomogram has the potential to improve diagnostic confidence, optimize referral pathways, and contribute to more personalized patient management in dermatology and primary care practice.

## 5 Conclusion

In conclusion, this retrospective analysis demonstrates that the combination of HFUS and SMI provides a valuable non-invasive approach for differentiating BCC from SK. The study identifies several independent ultrasound and clinical predictors, including larger tumor size, head location, nodular ultrasound pattern, and random vascular flow pattern, which collectively enhance diagnostic accuracy. The developed nomogram, integrating these key variables, exhibits good discriminative performance with an area under the curve of 0.861, offering a practical tool to estimate individualized probability of malignancy in clinical settings. These findings support the utility of multimodal ultrasound imaging as an adjunct to traditional diagnostic methods, potentially reducing unnecessary biopsies and facilitating earlier detection of malignant lesions. While the results are promising, further prospective validation across diverse populations is warranted to confirm generalizability and refine the model. Ultimately, this study contributes to the growing evidence for non-invasive imaging in dermatology and underscores the potential of ultrasound-based nomograms to aid clinical decision-making and optimize patient management.

## Data Availability

The original contributions presented in the study are included in the article/supplementary material, further inquiries can be directed to the corresponding author.

## References

[1] WHO Classification of Tumours Editorial Board. WHO Classification of Skin Tumours. 5th ed. Lyon: International Agency for Research on Cancer (2025). p. 58–76.

[2] Levit S Shoykhet J Levit E. Comprehensive insights into basal cell carcinoma: causes, presentation, prevention, and modern therapeutic approaches. Cancer Med. (2025) 14:e71448. doi: 10.1002/cam4.71448, 41416412 PMC12715586

[3] Barthelmann S Butsch F Lang BM Stege H Großmann B Schepler H. Seborrheic keratosis. J Dtsch Dermatol Ges. (2023) 21:265–77. doi: 10.1111/ddg.14984 36892019

[4] Morsia S Pizzichetta MA Kaleci S Chester J Ciardo S Alma A . Dermoscopy and reflectance confocal microscopy improves accuracy in differentiating atypical basal cell carcinoma from seborrheic keratosis and vice versa. Dermatology. (2025) 241:453–62. doi: 10.1159/000547590, 40774163

[5] Mei LH Cao MK Li J Ye XG Liu XD Yang G. Deep learning in assisting dermatologists in classifying basal cell carcinoma from seborrheic keratosis. Front Oncol. (2025) 15:1507322. doi: 10.3389/fonc.2025.1507322, 40342818 PMC12058839

[6] Boostani M Bozsányi S Suppa M Cantisani C Lőrincz K Bánvölgyi A . Novel imaging techniques for tumor margin detection in basal cell carcinoma: a systematic scoping review of FDA and EMA-approved imaging modalities. Int J Dermatol. (2025) 64:287–301. doi: 10.1111/ijd.17496, 39358676 PMC11771686

[7] Pequeno ALV Bagatin E. Dermatological ultrasound in assessing skin aging. Front Med (Lausanne). (2024) 11:1353605. doi: 10.3389/fmed.2024.1353605, 38410749 PMC10895009

[8] Dhami A Vale SM Richardson ML Schachtel AK Fleckman P. Comparing ultrasound with magnetic resonance imaging in the evaluation of subungual glomus tumors and subungual myxoid cysts. Skin Appendage Disord. (2023) 9:262–7. doi: 10.1159/000530397, 37564693 PMC10410070

[9] Álvarez-Salafranca M Gómez-Martín I Bañuls J Serrano P Medina C Llambrich A . Dermoscopy of inflamed seborrheic keratosis: a great mimic of malignancy. Australas J Dermatol. (2022) 63:53–61. doi: 10.1111/ajd.13781, 34958128

[10] Yuki A Takatsuka S Abe R Takenouchi T. Diagnostic accuracy of dermoscopy for 934 basal cell carcinomas: a single-center retrospective study. J Dermatol. (2023) 50:64–71. doi: 10.1111/1346-8138.16607, 36229917

[11] Karampinis E Georgopoulou KE Kampra E Zafiriou E Lallas A Lazaridou E . Clinical and dermoscopic patterns of basal cell carcinoma and its mimickers in skin of color: a practical summary. Medicina (Kaunas). (2024) 60:1386. doi: 10.3390/medicina60091386, 39336428 PMC11434363

[12] Wortsman X. Ultrasound in skin Cancer: why, how, and when to use it? Cancers (Basel). (2024) 16:3301. doi: 10.3390/cancers16193301, 39409920 PMC11475754

[13] Crisan D Wortsman X Catalano O Badea R Kastler S Badea A . Pre-operative high-frequency ultrasound: a reliable management tool in auricular and nasal non-melanoma skin cancer. J Dtsch Dermatol Ges. (2024) 22:357–65. doi: 10.1111/ddg.15308, 38243870

[14] Luo H Yin L. Diagnostic value of superb microvascular imaging and color Doppler for thyroid nodules: a meta-analysis. Front Oncol. (2023) 13:1029936. doi: 10.3389/fonc.2023.1029936, 37091165 PMC10113672

[15] Jasionyte G Seskute G Rugiene R Butrimiene I. Assessing scleroderma patterns with superb microvascular imaging: is it possible? New prospects for ultrasound. Clin Rheumatol. (2023) 42:301–6. doi: 10.1007/s10067-022-06405-7, 36214919

[16] Chen X Zhou L Xia Y Wong YN He Q Tang P . Superb microvascular imaging for evaluating the activity of juvenile localised scleroderma: a preliminary study. Eur Radiol. (2024) 34:6376–83. doi: 10.1007/s00330-024-10738-z, 38652159 PMC11399200

[17] Siskou S Pasquali P Trakatelli M. High frequency ultrasound of basal cell carcinomas: ultrasonographic features and histological subtypes, a retrospective study of 100 tumors. J Clin Med. (2023) 12:3893. doi: 10.3390/jcm12123893, 37373588 PMC10299541

[18] Cao L Cao Y Wang X Lu X Zhao F Sun L . Analysis of features of papillary thyroid carcinoma on color Doppler ultrasound images: implications for lymph node metastasis. BMC Med Imaging. (2025) 25:75. doi: 10.1186/s12880-025-01615-2, 40050763 PMC11883915

[19] Roland N Memon A. Non-melanoma skin cancer of the head and neck. Br J Hosp Med (Lond). (2023) 84:1–10. doi: 10.12968/hmed.2021.0126 37127417

[20] Wojtowicz I Żychowska M. Dermoscopy of basal cell carcinoma part 2: dermoscopic findings by lesion subtype, location, age of onset, size and patient phototype. Cancers (Basel). (2025) 17:176. doi: 10.3390/cancers17020176, 39857958 PMC11764052

[21] Crisan D Kastler S Scharffetter-Kochanek K Crisan M Schneider LA. Ultrasonographic assessment of depth infiltration in melanoma and non-melanoma skin cancer. J Ultrasound Med. (2023) 42:1609–16. doi: 10.1002/jum.16180, 36714967

[22] Crisan D Schneider LA Scharffetter-Kochanek K Bernhard L Crisan M Wortsman X. The usefulness of ultrasonography for supporting the differentiation, diagnosis, and treatment of atypical fibroxanthoma and pleomorphic dermal sarcoma. J Ultrasound Med. (2024) 43:1563–72. doi: 10.1002/jum.16478, 38703399

[23] Shen Y Wang R Zhao C Liu L Sun D Chen X. Investigations on ultrasonography in the diagnosis of nodular localized cutaneous neurofibroma. J Clin Ultrasound. (2024) 52:359–67. doi: 10.1002/jcu.23639, 38264918

[24] Oganesyan R Tahan SR. Colloid bodies in cutaneous basal cell carcinoma: clinical and histologic correlates-an analysis of 405 cases. J Cutan Pathol. (2026) 53:108–11. doi: 10.1111/cup.14755, 39608841

[25] Zhang S Zhu QL Xiao MS Liu J. The value of dermoscopy and high-frequency ultrasound in staging morphea. J Dermatol. (2023) 50:511–7. doi: 10.1111/1346-8138.16648, 36420557

[26] Dong B Xia H Liu Y Wang S Ye Z. Very-high-frequency ultrasonographic profiling characteristics of nodular hidradenoma: a retrospective analysis. Int J Gen Med. (2025) 18:5127–35. doi: 10.2147/IJGM.S534241, 40927776 PMC12416390

[27] Stawarz K Galazka A Misiak-Galazka M Durzynska M Gorzelnik A Bienkowska-Pluta K . Advances in skin ultrasonography for malignant and benign tumors of the head and neck: current insights and future directions. J Clin Med. (2025) 14:2298. doi: 10.3390/jcm14072298, 40217748 PMC11989985

[28] Lin TL Lee KH Karmakar R Mukundan A Sundarraj J Lu CT . Artificial intelligence-assisted dermatologic screening: epidemiology and clinical features of basal cell carcinoma, squamous cell carcinoma, seborrheic keratosis and actinic keratosis. Bioengineering (Basel). (2025) 12:1258. doi: 10.3390/bioengineering12111258, 41301214 PMC12650624

[29] Ma YY Gong XH Wang Q Wang LF Xu HX Guo LH. High-frequency ultrasound for evaluation of the pathological invasion level of extramammary Paget disease. J Ultrasound Med. (2022) 41:389–400. doi: 10.1002/jum.15716, 33856069

[30] Corvino A Varelli C Cocco G Corvino F Catalano O. Seeing the unseen with superb microvascular imaging: ultrasound depiction of normal dermis vessels. J Clin Ultrasound. (2022) 50:121–7. doi: 10.1002/jcu.23068 34761407

[31] Zou R Lin Y Da C Liao G. Novel nomogram and decision curve analysis for predicting head and neck skin cancer risk. Sci Rep. (2025) 15:41555. doi: 10.1038/s41598-025-25427-0, 41286108 PMC12644855

[32] Oh Y Zheng Z Kim KY Xu X Pei M Oh B . A nomogram combining clinical factors and biomarkers for predicting the recurrence of high-risk cutaneous squamous cell carcinoma. BMC Cancer. (2022) 22:1126. doi: 10.1186/s12885-022-10213-2, 36324094 PMC9632077

[33] Liu W Qu X Yan Y Li X Zhang Z Huang Y . A nomogram for predicting benign and malignant skin tumors using multimodal ultrasound. Ultrason Imaging. (2026) 48:26–35. doi: 10.1177/01617346251374629, 41486742

[34] Feng MC Liang JF Chen YX Lu JM Wang J. Construction of an aggressiveness diagnostic model for basal cell carcinoma using multimodal ultrasound features. J Ultrasound Med. (2025) 44:1839–48. doi: 10.1002/jum.16734, 40461439

[35] Li JM Shao YH Sun XM Shi J. Ultrasonic features of automated breast volume scanner (ABVS) and handheld ultrasound (HHUS) combined with molecular biomarkers in predicting axillary lymph node metastasis of clinical T1-T2 breast cancer. Quant Imaging Med Surg. (2024) 14:1359–68. doi: 10.21037/qims-23-956, 38415107 PMC10895107

[36] Jia K Li H Wu X Xu C Xue H. The value of high-resolution ultrasound combined with shear-wave elastography under artificial intelligence algorithm in quantitative evaluation of skin thickness in localized scleroderma. Comput Intell Neurosci. (2022) 2022:1613783. doi: 10.1155/2022/1613783, 35281193 PMC8916868

[37] Koppa BM Kelly CT. Point-of-care ultrasound in skin and soft tissue infections. J Hosp Med. (2024) 19:938–44. doi: 10.1002/jhm.13467, 39082276

